# Identification of Down-Regulated Proteome in *Saccharomyces cerevisiae* with the Deletion of Yeast Cathepsin D in Response to Nitrogen Stress

**DOI:** 10.3390/microorganisms7080214

**Published:** 2019-07-24

**Authors:** Jingjin Hu, Lingxiao Yu, Qin Shu, Qihe Chen

**Affiliations:** Department of Food Science and Nutrition, Zhejiang University, Hangzhou 310058, China

**Keywords:** *Saccharomyces cerevisiae*, yeast cathepsin D (Pep4p), proteomics, glycolytic enzymes, cellular metabolism

## Abstract

Vacuolar proteinase A (Pep4p) is required for the post-translational precursor maturation of vacuolar proteinases in *Saccharomyces cerevisiae*, and important for protein turnover after oxidative damage. The presence of proteinase A in brewing yeast leads to the decline of beer foam stability, thus the deletion or inhibition of Pep4p is generally used. However, the influence of Pep4p deletion on cell metabolism in *Saccharomyces cerevisiae* is still unclear. Herein, we report the identification of differentially down-regulated metabolic proteins in the absence of Pep4p by a comparative proteomics approach. 2D-PAGE (two-dimensional polyacrylamide gel electrophoresis) presented that the number of significantly up-regulated spots (the Pep4p-deficient species versus the wild type) was 183, whereas the down-regulated spots numbered 111. Among them, 35 identified proteins were differentially down-regulated more than 10-fold in the Pep4p-deficient compared to the wild-type species. The data revealed that Pep4p was required for the synthesis and maturation of several glycolytic enzymes and stress proteins, including Eno2p, Fba1p, Pdc1p, Tpi1p, Ssa1, Hsp82p, and Trr1p. The transcription and post-translational modifications of glycolytic enzymes like Eno2p and Fba1p were sensitive to the absence of Pep4p; whereas the depletion of the *pep4* gene had a negative impact on mitochondrial and other physiological functions. The finding of this study provides a systematic understanding that Pep4p may serve as a regulating factor for cell physiology and metabolic processes in *S. cerevisiae* under a nitrogen stress environment.

## 1. Introduction

*Saccharomyces cerevisiae* is one of the most widely used species; the cellular physiology and metabolism of this species are closely related to the quality and flavor of many fermented foods and drinks. Maintaining beer foam stability is a major challenge faced by beer manufacturers. Many factors affect beer foam stability, and as such, a systematic approach is required to address this issue. Yeast proteinase A (Pep4p) is regarded as a chief regulator that destroys foam protein, and especially can hydrolyze lipid transport protein (Ltp1), which is the major protein for stabilizing foam in sparkling wine and beer [1]. Recent studies have focused on gene knock-out examinations, such as deleting the *pep4* gene in brewing yeast and inserting the *pep4* locus with other expression cassettes like the *bgls* gene, *ltp1*, or PGK1p-ILV5-PGK1t expression cassette [2,3,4,5]. However, the use of transgenic yeast in brewing process is not widely accepted. Therefore, investigation of the key role of Pep4p in *S. cerevisiae* is needed in order to ultimately realize the precise regulation of fermentation processes at the genetic level.

Pep4p is essential to proteolytic systems in *S. cerevisiae*, and is implicated in the activities of other vacuolar hydrolases that include proteinase B (Prb1p), caboxypeptidase Y (Prc1p), and aminopeptidase I (Ape1p) [6,7]. This protease is vital for protein degradation, differentiation, and cell survival [8]. Recent studies have been conducted to examine the unique and novel biological functions of Pep4p in *S. cerevisiae*. Spedale et al. discovered that Pep4p is the protease responsible for SAGA-related (Spt-Ada-Gcn5-acetyltransferase complex-related) Slik protein complex formation [9]. As a RNA polymerase II transcription co-activator, the *S. cerevisiae* SAGA protein complex manifests different activities, including acetylation and deubuiqitination of histones and recruitment of TATA-binding proteins to promoters [9,10]. Apart from proteolytic activity, Pereira et al. subsequently uncovered a role for vacuolar protease Pep4p in mitochondrial degradation, using genetic approaches [11]. Depletion and overexpression of the *pep4* gene delayed and enhanced mitochondrial degradation, respectively. In addition, Marques et al. also revealed that Pep4p is important in protein turnover after oxidative damage [12]. Though the structure and functions of Pep4p in *S. cerevisiae* have been partially investigated, the systematic effect of this proteinase on cellular physiology and metabolic processes is unclear.

In a previous study, Zhang et al. showed that partial deletion of the *pep4* gene in industrial *S. cerevisiae* caused an extension in the lag phase of cell growth [4]. Further investigations characterized the influences of Pep4p on cell growth and carbon metabolism in industrial *S. cerevisiae* WZ65, and found that the lag phase of cell growth in Pep4p-deficient mutants was postponed when compared to the wild-type one [13]. Chen et al. suggested that Pep4p in industrial *S. cerevisiae* directly or indirectly affects the expression of glycolytic enzymes hexokinase (HK), phosphofructokinase (PFK), and pyruvate kinase (PK), and thus resulted in a delay in cellular metabolism [14]. Furthermore, the deletion of the *pep4* gene in industrial *S. cerevisiae* WZ65 not only affected PK synthesis, but also modulated the flux of oxidative phosphorylation [15]. Therefore, we assumed that the central carbon metabolism in *S. cerevisiae*, especially glycolytic fluxes (including EMP, TCA, PEK, etc.), is mediated by vacuolar Pep4p at the gene or protein level. The vacuolar protease Pep4p, an orthologue of human CatD, is released from the vacuole into the cytosol in response to stress [16,17]. It is proposed that there is a vacuole–mitochondrial cross-talk during apoptosis in yeast [18], but we still have no clear understanding of the metabolic link between mitochondria and vacuoles. In this work, the scientific truth we needed to reveal was the comprehensive influence underlying the downstream regulatory pathway of Pep4p. As a consequence, we used haploid *S. cerevisiae* as the microbial model, which has a relatively simple genetic background compared to industrial strain *S. cerevisiae*, and which should prove more beneficial to the evaluation of vacuolar Pep4p function. We utilized the strategies of proteomics and RT-qPCR to examine the regulatory influences of Pep4p on the central carbon metabolism of the yeast cell, to elucidate the systematic functions of Pep4p in haploid *S. cerevisiae*.

## 2. Materials and Methods

### 2.1. Yeast Strains and Chemostat Cultivation Conditions

*S. cerevisiae* strains BY4741 (MATa, his3Δ1, leu2Δ0, met15Δ0, ura3Δ0), and BY4741 Pep4Δ (the mutant containing the knock-out of the *pep4* gene encoding proteinase A) were obtained from Thermo Fisher Scientific Inc (Waltham, MA, USA). The deletion of Pep4p in the wild-type strain was identified by PCR amplification and DNA sequence analysis. The experimental strains were selected on G418 plates and grown until the absorbance of 1.0 at 600 nm (A600) in liquid yeast peptone dextrose (YPD) medium containing 1% yeast extract, 2% peptone, and 2% glucose at 30 °C with shaking at 180 rpm.

The haploid *S. cerevisiae* strains (BY4741 and the Pep4p-deficient mutant) were grown at 28 °C in YPD medium. The seed culture was inoculated with well-grown yeast from an agar slant and incubated in a test tube containing 5 mL YPD liquid medium for 12 h. The seed culture broth was then inoculated into flasks containing 300 mL specific fermentation medium. In order to compare physiological indices of the two strains at the same growth stage, we conducted glucose-limited chemostat cultivation. The specific culture medium contained half the concentration of glucose, twice the yeast extract, and the peptone concentration of normal medium. Glucose was added during the submerged cultivation period to ensure that the concentration of remaining reduced sugar in the culture broth was above 1 mg/mL. After culturing for 15 h, yeast cells were transferred to YPD or SD (Synthetic Dropout) medium (0.67% (*w*/*v*) yeast nitrogen base, 2% (*w*/*v*) glucose), and the rotation rate was controlled at 120 rpm at 28 °C. The cultured cells were sampled at different times and collected for the determinations shown below.

### 2.2. Preparation of Intracellular Protein Extracts

Cultured cells of *S. cerevisiae* were washed three times with ultrapure water and collected by centrifugation at 3000× *g* for 15 min at 4 °C. The collected cells were added into protein extraction buffer containing 1% (*w*/*v*) SDS (sodium dodecyl sulfate), 50 mM Tris, pH 8.0, 5 mM EDTA (Ethylenediaminetetraacetic acid disodium salt), and 1 mM phenylmethylsulfonyl fluoride after lyticase treatment, then were placed on wet ice and homogenized at 11,000× *g* for 2 s with 12 iterations. After incubation at 4 °C for 1 h, the samples were centrifuged at 4000× *g* for 1 h, and the supernatants were collected. Total protein concentrations of the samples were assayed using a Bradford-based protein assay (Bio-Rad, Hercules, CA, USA), using bovine serum albumin (BSA) as a standard [19]. For the proteomic analyses, triplicate protein extracts from three independent cultures were individually prepared for the 2-DE (two-dimensionalelectrophoresis) experiment.

### 2.3. SDS-PAGE Gel Electrophoresis

Yeast cells were cultivated in rich medium (YPD) and minimal medium (SD) to assess the intracellular protein profiling in complete nutritional and nitrogen starvation conditions, respectively. The intracellular proteins of yeast cells cultured in both media at different times were examined by vertical SDS-PAGE using 12% resolving gel [20]. Measures of 10 μL of the reaction mixture were loaded into each lane and proteins were stained with Coomassie Brilliant Blue R-250 after gels were fixed with 10% (*w*/*v*) trichloroacetic acid [21]. According to the result in SDS gel electrophoresis, YPD medium was chosen for proteomic analysis to avoid interference of poor nutritional status.

### 2.4. Two-Dimensional Polyacrylamide Gel Electrophoresis

For isoelectric focusing (IEF), samples containing proteins were dissolved in rehydration buffer consisting of 8 M urea, 2% (*w*/*v*) 3-[(3-cholamidopropyl) dimethylammonio]-1-propanesulfonate (CHAPS), 65 mM dithiothreitol (DTT), and bromophenol blue. The total volume of the sample and the rehydration buffer was 450 μL, and this was added to Ready Strip immobilized pH gradient (IPG) strips. IEF was carried out under the following conditions: 6 h at 30 V, 6 h at 60 V, 1 h at 500 V, 1 h at 1000 V, and 20 h at 8000 V. At 64,000 Vhr, the IEF was terminated and the proteins were subsequently separated in the second dimension on the basis of their molecular mass. The strip was applied to a 12.5% SDS-PAGE gel, and electrophoresis was performed at 4 W per gel for 45 min, and then at 15 W per gel until the bromophenol blue dye front reached the bottom of the SDS-PAGE gel. After the electrophoresis step, protein spots were visualized by staining with silver nitrate and then scanned to detect protein spots on the 2-DE gel. Image Master TM 2D Platinum software Version 5.0 (Amersham Biosciences, Piscataway, NJ, USA) was used to quantify the volume of each spot. The computer-assisted PD-Quest gel analysis software (Bio-Rad, Hercules, CA, USA) was used to perform qualitative and quantitative analysis of the differentially expressed proteins. Those proteins isolated on 2-DE gels that showed repeatability in at least three paired samples, and expression differences beyond a 10-fold change, were selected for MALDI-TOF MS analysis. Student’s t test (*p* < 0.05) was used to determine whether the relative changes in protein abundance were statistically significant.

### 2.5. Mass Spectrometry Analysis and Database Search

After the 2-DE step, the selected spots were identified by mass spectrometry, as previously described [22]. Briefly, the selected protein spots were cut from the 2-DE gels and digested with trypsin. The excised spots were extracted by sonication, using ddH_2_O. The identified spots were then dehydrated in acetonitrile and dried in a speed vacuum centrifuge for 5 min. Subsequently, in-gel digestion of the excised proteins was carried out by adding 2–3 µL 10 ng/µL trypsin solution in 25 mM NH_4_HCO_3_ to the gel pieces, and the samples were incubated overnight at 37 °C for 12 h. The process of digestion was terminated by adding 0.1% trifluoroacetic acid (TFA), and the samples were centrifuged. A total 1 µL of the desalted sample was mixed with 1 µL matrix and spotted onto the MALDI target. Samples were air-dried at room temperature and analyzed using Autoflex II MALDI-Q-TOF (matrix-assisted laser desorption/ionization quadrupole time-of-flight) tandem mass spectrometry (Bruker Daltonics, Bremen, Germany). Peptide mass maps were acquired in positive reflection mode, averaging 800 laser shots per MALDI-TOF spectrum and 800 shots per TOF/TOF spectrum. Resolution was 15,000–20,000. The Bruker calibration mixtures were used to calibrate the spectrum to a mass tolerance within 0.1 Da. Each acquired mass spectrum (*m*/*z* range 700–4000) was processed using the software Flex Analysis v. 2.4 (Bruker Daltonics, Bremen, Germany). The peak detection algorithm was SNAP (Sort Neaten Assign and Place); S/N threshold, 3; quality factor threshold, 50. The tryptic autodigestion ion picks (842.51 Da and 2211.10 Da) were used as internal standards to validate the external calibration procedure. Matrix and/or autoproteolytic trypsin fragments and known contaminant ions, such as keratins, were excluded. The resulting peptide mass lists were used to search the IPI (Inter-national Protein Index) rat sequence database (http://www.ebi.ac.uk/IPI/, version 3.31; 41251 sequences; 21545744 residues). The following search parameter criteria were used: significant protein MOWSE score at *p* < 0.05, minimum mass accuracy 100 ppm, trypsin as the enzyme, 1 missed cleavage site allowed, cysteine carbamidomethylation, acrylamide modified cysteine, methionine oxidation and similarity of pI and relative molecular mass specified, and minimum sequence coverage of 15%.

The mass spectra were obtained on the MALDI-Q-TOF-MS/MS system. The identification of the peptide mass fingerprints was carried out using MASCOT (http://www.matrixscience.com). Information on the identified proteins was referenced from the *Saccharomyces* Genome Database (http://www.yeastgenome.org/), from the Comprehensive Yeast Genome Database-Munich Information Center for Protein Sequences databases (http://mips.helmholtz-muenchen.de/genre/proj/yeast/), and from the Kyoto Encyclopedia of Genes and Genomes (http://www.genome.jp/keg/pathway.html) for reconstructing major metabolic pathways.

### 2.6. Real-Time Quantitative PCR (RT-qPCR) Assay

The three enzymes identified as having the largest fold-changes, which are all involved in carbohydrate metabolism, were selected to further examine their relevant gene expression by RT-qPCR using the ABI Prism 7700 Sequence Detection System (Perkin-Elmer Applied Biosystems, Foster City, CA). The experimental procedure was as follows. RNA extraction from the yeast cells was carried out using Trizol reagent and chloroform. First, 1 mL Trizol was added to yeast cells for about 5 min at room temperature. Next, chloroform was added and the suspension was vortexed for 15 seconds. After 3 min, the liquid culture broth was centrifuged at 8000× *g* for 10 min at 4 °C. The supernatant was then extracted again with 0.5 mL isopropyl alcohol on wet ice for 20 to 30 min. After a second identical centrifugation step, 1 mL of 75% ethanol was added, and then the precipitate was washed and centrifuged. Total RNA was extracted with RNase-free H_2_O. The quality and concentration of RNA were assessed spectrophotometrically and by electrophoresis in 1% agarose. For the RT step, cDNA was synthesized from 5 μL total RNA using Prime Script Reverse Transcriptase (Takara, Kyoto, Japan) as the RT enzyme. The cDNA was frozen at −80 °C. Real-time quantitative PCR was performed in 96 well plates on an ABI7500 FAST instrument (Applied Biosystems, Foster City, CA, USA) using the SYBR Green stain that binds double-stranded DNA [23]. Reactions were carried out in a total volume of 25 μL, containing 2 μL of cDNA, 1 μM of forward and reverse primers, and 12.5 μL of 2×SybrGreen qPCR Master Mix. A non-template control for each primer was included in all real-time plates. Amplifications were performed under the following conditions: 95 °C for 15 min; 40 cycles of 95 °C for 10 s, and 60 °C for 40 s. At the end of the amplification cycle, a melting analysis was conducted to verify the specificity of the reaction. All samples were analyzed in triplicate. The expression levels were described in terms of the cycle threshold value (Cq), which was the number of cycles required to reach a certain fluorescence value (threshold) [24]. Threshold values were obtained using the automated setting of the instrument software (base line-subtracted curve-fit data), and considered the fluorescence when the maximal efficiency of PCR was achieved. The data expressed as Cq were imported into a Microsoft Excel data sheet for subsequent analysis. We used the 2−ΔΔCT method to analyze relative gene expression levels [25].

The primers used in this work were the following: actin1-F(5′-ACTTTCAACGTTCCAGCCTTC-3′) and actin1-R (5′-CGTAAATTGGAACGACGTGAGTA-3′) for YFL039C/actin1; fba1-F (5′-AGG AACACGGTGAACCATTATT-3′) and fba1-R (5′-TACCGATTTCCATTTCTAACCA-3′) for YKL06 0C/fba1; eno2-F (5′-CGTCAAGGCCAACCTAGATGT-3′) and eno2-R (5′-CCAAATGTTGGTACA ATGGGAC-3′) for YHR174W/eno2; pdc1-F (5′-TTAAGGAAGCCGTTGAATCTG-3′) and pdc1-R (5′-TGATCTTCATGTGGTCGGAGT-3′) for YLR044C/pdc1.

## 3. Results

### 3.1. Pep4p Proteolyticeffect on Intracellular Proteins and Cell Growth under Different Culture Conditions

**Figure 1:**
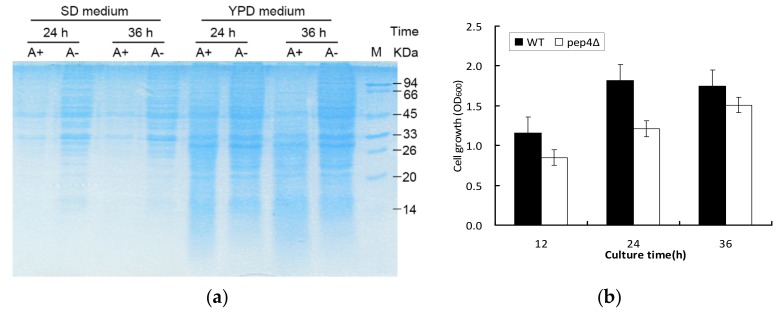
Cell growth and intracellular proteins expression under glucose-limited chemostat cultivation between BY4741 (A+) and pep4Δ one (A−). (**a**) SDS-PAGE showing the intracellular protein profile of BY4741 (A+) and pep4Δ strain (A−) cultured in SD and YPD medium at different culture times, respectively; (**b**) cell growth with the absorbance of 600 nm.

To investigate the effect of proteolytic activity of Pep4p, SDS-PAGE was performed in both complete nutritional (YPD medium) and nitrogen starvation conditions (SD medium) (Figure 1a. The profiles of the first four samples (Figure 1, lane 1–4) extracted in SD medium clearly indicated that a large number of intracellular proteins were preserved in the pep4Δ species (A−) compared with the wild-type BY4741(A+) when cultured for 24 h and 36 h. Although the proteins at 33 and 45 kDa were obviously observed in both yeast strains, the presence of Pep4p could lead to the reduction of these proteins. The comparable disparity was apparent in YPD medium, despite producing more proteins (Figure 1, lane 5–8). Cell growth was comparatively examined between BY4741 (A+) and the Pep4Δ species (A−). Figure 1b shows that the pep4Δ possessed the postponed lag phase in contrast to the control, and its cell growth was clearly rather slower than that of the control. The findings indicate that Pep4p plays a central role in the intracellular proteolysis pathway and primary metabolism. Plenty of proteins would be degraded by Pep4p, but of even greater concern is the down-regulated proteins affected by the knockout of Pep4p. We need to understand the correlation between *Pep4* gene and overall metabolism regarding cell growth and stress response.

### 3.2. 2-DE Profiling of Wild-Type S. cerevisiae and Pep4p-Deficient Strain

**Figure 2:**
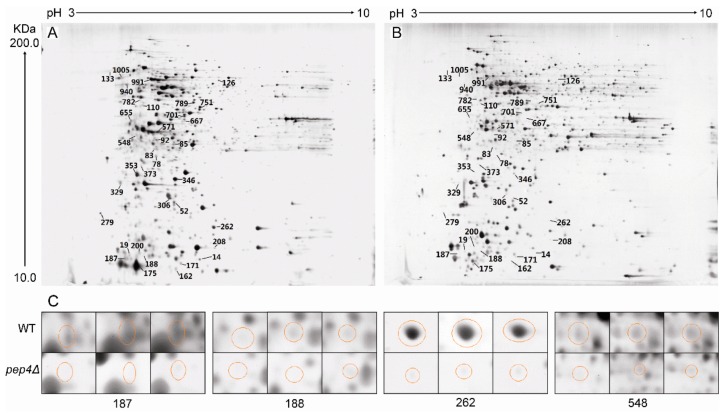
2-DE gel maps of intracellular proteins from BY4741 (WT) and pep4Δ strain. (**A**) wild-type strain BY4741. (**B**) Pep4p-deficient yeast strain (pep4Δ). (**C**) Ssa1 (Spot 187), Fba1p (Spot 188), Eno2p (Spot 262), and Pdc1p (Spot 548) from three independent 2-DE gel maps were compared, and highlighted with circles.

2-DE images of the wild-type (WT) and the Pep4p-deficient (Pep4Δ) are presented in Figure 2A,B, respectively. Silver staining of proteins from the WT and the mutant (with a matching rate of 88.9%), averaged 1163 ± 19 and 1162 ± 26 spots, respectively (Figure 2). We determined that the number of significantly up-regulated spots (i.e., from the Pep4p-deficient strain with respect to the WT) was 183, whereas the down-regulated spots numbered 111. Differentially expressed proteins were defined as being statistically significant based upon their differences in intensity (the ratio of the WT versus the pep4Δ one). It was considered significant in this image analysis when the proteins exhibited more than a two-fold change. Many different proteins were seen in abundance between the two designated species. Among these, 35 significantly different spots were determined (based upon a ratio between abundance of the wild-type to the Pep4p-deficient cells above 10 (t-test, p < 0.01)) to be further identified by MALDI-Q-TOF-MS/MS.

#### 3.2.1. Identification of Down-Regulated Proteins and Gene Ontology (GO) Analysis

Through MS analysis, 35 proteins were definitively identified. The abundances of all these 35 proteins were decreased compared to the WT. Descriptions of these proteins, including their accession numbers in NCBI, molecular properties, fold-changes and putative biological functions, are depicted in Table 1. We also analyzed the results by Blast2 GO V.2.5.0. We first analyzed the cellular components of the yeast proteins and found annotations for 35, comprising 114 different GO categories. After fusing the detailed categories into more general ones, we found four major terms showing different cellular components. A high proportion of the identified proteins were localized in the cytoplasm and organelle. In the “cellular components” part, functional reactions identified with a high protein concentration were categorized into “cell part,” “membrane-bound organelle,” and “non-membrane bound organelle”. In addition to cellular components, the software enabled us to identify the molecular functions and biological processes of the identified proteins. The leading functions with high protein concentration in the molecular function part were involved in protein binding, nucleic acid binding, and hydrolase activity. We also analyzed annotations from the branch biological process of GO. Similarly, cellular metabolic processes, primary metabolic processes, and macromolecule metabolic processes had an expected higher protein distribution in the changed biological processes for Pep4p deficiency in *S. cerevisiae.* Many down-regulated proteins were also involved in nitrogen compound processes.

#### 3.2.2. Identification of Key Down-Regulated Proteins Involved in Yeast Cell Metabolic Pathways

Table 1 shows that the identified proteins were classified into different groups according to their biological functions. The most abundant proteins found were involved in protein processing in endoplasmic reticulum (Ssa1 and Hsp82p), glycolysis/gluconeogenesis (Eno2p, Pdc1p, Tpi1p, and Fba1p), intracellular transport (Arl1p, Kha1p, and Nup170 -like protein), transcriptional regulation (Ino4p, Lsm6p, Prp18p, Dbp2p, Aep3p, and SCY_5111), and amino acid metabolism (Mks1p, Gsh1p, and Aro1p). Other proteins participated in protein phosphorylation (Rts3p)/dephosphorylation(Ypk2p), mating response pathway (Scp160p), nucleotide excision repair (Rad14p), heme biosynthetic pathway (Hem14p), pyrimidine metabolism (Trr1p), inositol phosphate metabolism (Pik1p), and oxidative phosphorylation (Ppa2p).

**Figure 3:**
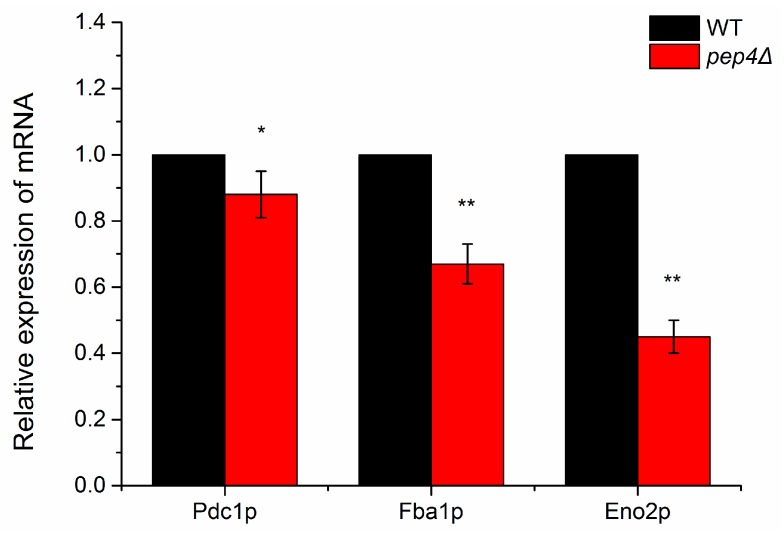
Relative mRNA expression levels of three identified down-regulated glycolytic metabolism enzymes in pep4Δ strain compared with the BY4741 (WT). The mRNA expression of the wild-type strain is set to 1, and the data represent means ± S.D. (*n* = 3). * *p* < 0.05, ** *p* < 0.01 compared to BY4741.

Among these identified proteins, of particular interest was the group of enzymes involved in glycolysis and gluconeogenic processes, including Eno2p, Pdc1p, Fba1p, and Tpi1p (Figure 2). Herein, these spots with high scores were selected for further analysis, in spite of the fact that some different spots were identified as the same enzymes Eno2p (Spot 208 and 262) and Pdc1p (Spot 353 and 548). The mass spectra of these proteins are shown in Figure 3. Moreover, we measured the enzyme specific activity of three identified down-regulated glycolytic metabolism enzymes in pep4Δ strain comparing with the BY4741 (WT) and result showed in Figure S1. Spot 188 was Fba1p, which is involved in the glycolytic process catalyzing the breakdown of fructose 1,6-diphosphate into dihydroxyacetone phosphate and glyceraldehyde 3-phosphate. The knock-out of the *Pep4* gene significantly down-regulated the synthesis of Fba1p, thereby influencing the glycolysis process. Eno2p is an enolase of glycolysis. This enzyme catalyzes the conversion of glycerate 3-phosphate into enolpyruvate phosphate. 2-DE results revealed that the ratio of Eno2p abundance in the wild-type strain to Pep4p-deficient strain cells was 23.53 ± 9.15/44.79 ± 6.47; thus, the absence of the *pep4* gene exerted a significantly negative influence on the synthesis of Eno2p. Pdc1p catalyzes the breakdown of pyruvate into acetaldehyde and carbon dioxide, which is then followed by alcoholic fermentation. Spot 133 is triose phosphate isomerase (Tpi1p), which takes part in the conversion between glyceraldehyde-3P and glycerone-p. The hypothetical protein SCY_4911 (Spot 126), which participates in carbohydrate metabolic process, was also down-regulated by the deletion of the *pep4* gene.

**Figure 4:**
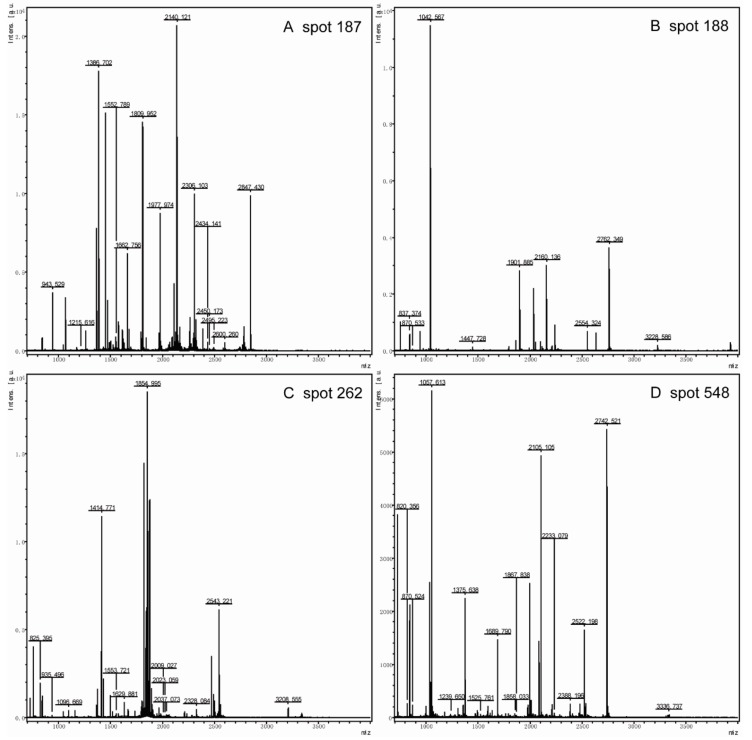
The mass spectrum of Spot 187 (**A**), Spot 188 (**B**), Spot 262 (**C**), and Spot 548 (**D**) in the 2-DE gel map of the wild-type yeast strain BY4741. Spot 187 was identified as Ssa1; Spot 188, Spot 262, and Spot 548 were identified as Fba1p, Eno2p, and Pdc1p, respectively.

On the 2-DE gel, Arl1p (Spot 92) is a soluble GTPase which plays a role in regulating membrane traffic. Kha1p (spot 701) plays a potent role in intracellular cation homeostasis, and Nup170 -like protein (Spot 782) is a structural constituent of the nuclear pore. Three proteins involved in intracellular transport were markedly down-regulated by the knock-out of the *pep4* gene. However, the most striking down-regulated protein was Ssa1p (Spot 187), the gene expression of which is influenced by both translational and post-translational processes; its mass spectrum is shown in Figure 4. Hsp82p (Spot 162) is the chaperone of Hsp90p, and increases in response to DNA replication stress. In addition, six down-regulated proteins participate in transcriptional regulation, including Ino4p (Spot 19), Lsm6p (Spot 78), Prp18p (Spot 110), Dbp2p (Spot 473), SCY_5111 (Spot 571), and Aep3p (Spot 655). The functional annotation of these six proteins is shown in Table 1. Moreover, Mks1p (Spot 667), Gsh1p (Spot 751), and Aro1p (Spot 1005) are important proteins in the biosynthesis of lysine, glutamylcysteine, and other aromatic amino acids, respectively. In addition, the mass spectrum of spot 187, 188, 208 and 548 in 2-DE gel map of the wild-type yeast strain BY4741 showed in Figure S1.

### 3.3. Verification of mRNA Levels of Key Down-Regulated Genes Relating to Glycolytic Flux

To further understand gene expression of the key enzymes, we evaluated those possessing greater mRNA abundance ratios between the wild-type and the mutant by RT-qPCR. Compared with the wild-type species, the mRNA levels of Eno2p, Fba1p, and Pdc1p in Pep4Δ one were clearly down-expressed after 24 h of culture (Figure 3). In this work, genes were identified as significantly differentially expressed by RT-qPCR when differences were ≥ 1.5-fold changes (*p*-value ≤ 0.05). Eno2p and Fba1p were significantly down-expressed in pep4Δ species compared to the WT one (*p* < 0.01). Based on the results of the 2-DE and MS analysis, we concluded that Pep4p regulates the transcriptional expression of Eno2 and Fba1; however, with respect to Pdc1p, the mRNA level in the mutant was lower than in the WT (*p* < 0.05), but Pep4p did not have a significant impact on Pdc1p at the transcription level. To analyze the activity of three investigated gylcolytic enzymes, the findings in Figure S2 demonstrated the same change as the mRNA data.

## 4. Discussion

*S. cerevisiae* is one of the most widely used microbes; its cellular physiology and metabolism are closely relevant to the quality and flavor of many fermented foods and drinks, such as bread, beer, and wine. Pep4p (yeast CatD) belongs to the class of pepsin-like aspartic proteases [6], which is localized predominantly in the vacuolar compartment; however, translocation of this protease from vacuole to the cytosol has also been described [18]. Pep4p is essential to the yeast life cycle, including protein degradation, differentiation, and cell survival [8,11,12]. This protease interferes with mitochondrial degradation [11], while the intracellular organelle is the most important power house for yeast cells. It has been suggested that Pep4p likely plays an important role in affecting energy metabolism, but few researchers have focused on the probable impact on glycometabolism by the knock-out of *pep4* gene in yeast. Recent studies have shown that Pep4p not only regulates cell endurance to stress shock, but also plays an important role in central cellular metabolism with respect to glycolytic flux in industrial *S. cerevisiae* [14,15]. The biological regulatory mechanism underlying these activities, however, remains to be uncovered. There is an urgent need to identify the novel proteins or signaling factors influenced by vacuolar protease Pep4p. The intracellular protein profile of wild-type BY4741 and Pep4p-deficient ones clearly indicates that Pep4p is important for protein degradation and turnover in *S. cerevisiae*. Thus, we adopted a comparative proteomic approach to annotate the differentially expressed proteins between the Pep4Δ and the wild-type yeast, with the aim to find unique, significantly changed proteins or regulators that may participate in the cell metabolism.

### 4.1. Glycolytic Metabolism is Influenced by Pep4p Deficiency

**Figure 5:**
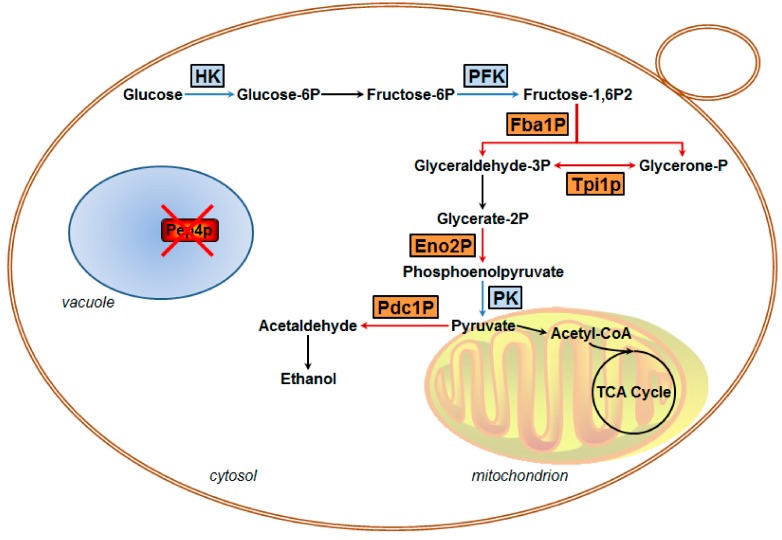
Putative overview of glycolytic metabolic processes in haploid *S. cerevisiae* regulated by vacuolar protease Pep4p based on proteomic analysis and RT-qPCR examination. Red cross represents Pep4p deletion. The blue frames show the proteins that participated in glycolysis in this work, while the orange frames show four identified down-regulated glycolytic enzymes involved in previous findings [14].

It is well acknowledged that Pep4p participates in the post-translational regulation of *S. cerevisiae* vacuolar hydrolases [26], but no relevant studies have focused on clarifying the correlation between vacuolar Pep4p and metabolic enzymes in central metabolism. In our previous research, it was found that the activities of HK, PFK, and PK were significantly lower in the Pep4p-deficient variant as compared to the wild-type [14]. It has been implied that the glycolytic pathway is influenced by the presence of vacuolar Pep4p in *S. cerevisiae*. The finding was further supported by the present data. Combined with the proteomics data and RT-qPCR quantifications, we concluded that vacuolar Pep4p influences the glycolysis/gluconeogenesis of haploid *S. cerevisiae* through a Pep4p-dependent pathway. The glycolytic pathway, as influenced by vacuolar Pep4p, is presented in Figure 5. The glycometabolic pathway map depicts four key proteins with large fold-changes by proteomic analysis (shown in orange); all are involved in the glycolytic flux of *S. cerevisiae*. Regarding Fba1p and Eno2p, they are involved in the glycolysis process. The reduced protein expression of these two enzymes could lead to a declining accumulation of pyruvate and consumption of fructose-1,6-bisphosphatase (Fructose-1,6P2). Thus, Pep4p deficiency led to rapid cell death when glucose-grown cells were starved for nitrogen or other nutrients, but catabolite inactivation and degradation of fructose-1,6P2 were not affected to a significant extent in the Pep4p-deficient cells [8]. As an important metabolic intermediate, not only can pyruvate be further metabolized through the TCA (tricarboxylic acid) cycle and via ethanol fermentation, it can also be further metabolized by anaerobic respiration, and by amino acid and fat metabolism. Therefore, it is apparent that the decreasing accumulation of pyruvate caused by the absence of vacuolar Pep4p might affect the overall metabolism of *S. cerevisiae* cells. Recently, it was reported that deletion of the *pep4* gene resulted in both apoptotic and necrotic cell death during chronological aging, but the prolonged overexpression of Pep4p extended chronological lifespan specifically through the protein’s anti-necrotic function [27]. As an important factor in primary metabolism during cellular death or aging process, we postulate that the glycolytic metabolism affected by Pep4p likely led to a series of physiological and biochemical reactions in *S. cerevisiae*. In contrast to the WT, the protein abundance and mRNA expression level of Eno2p and Fba1p were obviously down in the *pep4*-deleted mutant. However, this was not the same case for Pdc1p. With respect to the dramatically reduced protein abundance, the mRNA expression level of Pdc1p did not show a significant decline. It is possible that the post-translational process of Pdc1p is markedly influenced by vacuolar Pep4p, which remains to be elucidated in future study.

### 4.2. Pep4p Deletion Plays a Systematic Role in Cellular Physiology

Though more effort has been paid to investigating various functions of Pep4p in *S. cerevisiae*, they have been without systemic understanding of the influence Pep4p exerts on cell metabolic processes. Pep4p has been proved to be required for efficient mitochondrial degradation, but the exact mechanism underlying the downstream regulatory pathway of Pep4p is unclear [11]. In this work, we found that expression of mitochondrial inorganic pyrophosphatase (Ppa2p) and phosphatidylinositol 4-kinase (Pik1p) was down-regulated in the Pep4p deletion strain compared with the WT. Ppa2p is required for mitochondrial function and is possibly involved in energy generation from inorganic pyrophosphate [28], and Pik1p may control nonselective autophagy and mitophagy through trafficking of Atg9p [29]. Mks1p, which is involved in retrograde (RTG) mitochondria-to-nucleus signaling [30], also decreased markedly in the Pep4p-deficient variant. Carmona-Gutiérrez et al. found that Pep4p played a dual cytoprotective function, composed of anti-apoptotic and anti-necrotic components [27]. Herein, the reduced Rad14p in *Pep4*Δ species provided a possible explanation; because of that this protein could recognize and bind damaged DNA during nucleotide excision repair [31]. In addition, both Pac2p and Ypk2p were down-regulated in the *Pep4*Δ strain. The former protein is required for normal microtubule function [32], while the latter participates in a signaling pathway required for optimal cell wall integrity [33]. Based on these discoveries, we propose that vacuolar Pep4p likely has a systematic effect on cellular physiology and metabolic processes.

In response to H_2_O_2_ and acetic acid shocks, Pep4p migrates from the vacuole into the cytosol [16,17], and its translocation is associated with an increase in vacuolar permeability. The latter is correlated with the degradation of nucleoporins, and results in an increase in nuclear pore complex permeability. Interestingly, the proteins participating in intracellular transport, like Arl1p, Kha1p, and Nup170 -like protein, were down-regulated more than 10-fold in Pep4Δ species compared with the WT. In addition, the biosynthesis of some amino acids, like lysine and glutamyl cysteine, was affected by Pep4p though the related proteins, including Mks1p, Gsh1p, Aro1p, and Pdc1p. However, further investigations are needed to elucidate the effects of up-regulated proteins on cell metabolism and physiology in response to the deletion of vacuolar Pep4p in *S. cerevisiae*.

### 4.3. Knockout of Pep4p Interferes in Post-Translational Modification and Transcriptional Regulation

Regulation of glycolysis/gluconeogenesis is not only exerted by expression of glycolytic genes and interactions of the glycolytic proteins within their environment, but also in post-translational modifications (PTMs) and transcriptional regulation. PTMs including protein phosphorylation and hydrolysis are increasingly considered to be key regulators in a majority of cellular processes. Most enzymes are regulated by PTMs, and PTMs provide one of the fastest ways by which cells can adjust to environmental cues and internal stimuli [34]. The difference between protein abundance and enzyme activity of the above three glycolytic enzymes is greatly due to regulation by PTMs. Rts3p, which is a putative component of the protein phosphatase type 2A complex, may be related to this process. In this study, Ino4p, Lsm6p, Prp18p, Dbp2p, Aep3p, Scp160p, SCY_5111, and Ndt80-like protein are possibly potential regulators of glycolytic enzymes that were down-regulated in absence of Pep4p. Thus, the exact pathway by which Pep4p exerts a regulatory role as an apoptotic and/or necrotic executioner, apart from its pro-survival roles, remains to be explored. Yeast appears to employ a variety of mechanisms to ensure functional robustness of the central metabolism. An uninterrupted flow of energy and precursor metabolites through the glycolytic pathway via EMP, protein phosphorylation (PPS), and TCA cycle is ensured by a variety of adaptive mechanisms.

### 4.4. Depletion of Pep4 Gene Negatively Impacts Stress Proteins Expression

Under stress conditions, like acetic acid treatment, chronological aging, and oxidative and nutritional stresses, the presence of Pep4p had a beneficial effect on cell growth and survivability [11,12,27]. But the effect of vacuolar *pep4* gene on the expression of stress proteins has been paid little attention. Kim et al. found that gene expressions of metabolic enzymes (Fba1p, Pgk1p, Eno2p, Tpi1p, and Adh1p), an antioxidant enzyme (Ahp1p), a molecular chaperone (Ssb1p), and pyrimidine biosynthesis-related enzyme (Ura1p) were up-regulated in transgenic yeast cells under oxidative stress [35]. The influence of cAMP-dependent protein kinase (PKA) on protein synthesis was investigated during exponential growth under osmotic stress, showing that Hxk2p, Pdc1p, Ssb1p, Met6p, Atp2p, and Hsp60p displayed a partially PKA-dependent repression [36]. Based on the proteomic analysis, Ssa1Ssa1, Hsp82p, and Trr1pwere identified as the stress-related proteins in response to *pep4* deletion. The fold change of Ssa1 abundance in the WT to the Pep4Δ one reached up to 68.05 ± 13.55. Heat-shock proteins of the 70 kDa family protect nascent polypeptides as they emerge from the ribosome, assist in the transport of proteins into the nucleus, mitochondria, and endoplasmic reticulum [37], and play important roles in either refolding damaged proteins or shepherding their ubiquitination and degradation. Trr1p functions in maintaining cellular redox states and defending against oxidative stress, and was found to increase in abundance when *Candida albicans* cells exposed to amphotericin B [38]. Clearly, the deletion of Pep4p leads to a significant decrease in stress protein expression by *S. cerevisiae*, but the regulatory mechanism remains to be elucidated.

## 5. Conclusions

In summary, this work indicates that Pep4p greatly influences glycolytic metabolism and stress responses in *S. cerevisiae.* The absence of vacuolar proteinase A exerted a dramatic effect on cell physiology and metabolic processes, verified at the proteomic level. All 35 identified proteins were down-regulated under the condition of Pep4p deficiency. Simultaneously, the transcription of two glycolytic enzymes Eno2p and Fba1p varied concomitantly with protein synthesis in the mutant after the knock-out of the *pep4* gene. These findings are important for understanding the Pep4p-dependent metabolic link in vacuole–mitochondrial cross-talk in *S. cerevisiae*, and may offer insights for ultimately seeking for the key to precise regulation of fermentation process.

## Figures and Tables

**Table 1:** Down-regulated proteins identified after knock-out of the *Pep4* gene in *S. cerevisiae.*

Spotno ^a^	Accession No. in NCBI	Protein Identity	Mr (kDa)/pI	Coverage (%)^b^	Fold Change (C/M)^c^	Protein Score	Function/Annotation
Glycolysis/Gluconeogenesis							
133	gi|6320255	Tpi1p	26.89/5.75	38	16.22 ± 1.81	74	Triose phosphate isomerase.
188	gi|6322790	Fba1p	39.88/5.51	67	11.53 ± 4.69	242	Fructose 1,6-bisphosphate aldolase.
208/262	gi|6321968	Eno2p	46.94/5.67	36/47	23.53 ± 9.15/44.79 ± 6.47	94/130	Enolase II, catalyzes conversion of 2-phosphoglycerate to phosphoenolpyruvate during glycolysis and the reverse reaction during gluconeogenesis.
353/548	gi|6323073	Pdc1p	61.69/5.80	36/53	40.91 ± 8.23/12.26 ± 1.64	91/159	Major of three pyruvate decarboxylase isozymes; decarboxylates pyruvate to acetaldehyde; involved in amino acid catabolism.
Intracellular transport							
92	gi|323349761	Arl1p	19.51/5.13	48	37.88 ± 5.01	150	Soluble GTPase with a role in regulation of membrane traffic; regulates potassium influx; Gprotein of the Ras superfamily, similar to ADP-ribosylation factor.
701	gi|323337062	Kha1p	68.02/5.05	33	33.39 ± 10.78	165	Putative K+/H+ antiporter with a probable role in intracellular cation homeostasis.
782	gi|207347902	Nup170-like protein	87.97/5.25	40	32.31 ± 9.57	278	Nucleocytoplasmic transport, structural constituent of nuclear pore.
Stress response proteins							
162	gi|110590736	Hsp82p	26.99/5.53	49	11.66 ± 4.18	190	Hsp90 chaperone; chain A, yeast Hsp82 in complex with the novel Hsp90 inhibitor 8-(6-bromo-benzo[1,3]dioxol-5-ylsulfanyl)-9-(3-isopropylamino-propyl)-adenine.
171	gi|6320560	Trr1p	34.45/5.69	31	11.92 ± 2.30	131	Cytoplasmic thioredoxin reductase.
187	gi|172713	Ssa1Ssa1	37.59/5.24	63	68.05 ± 13.55	181	70kDa heat shock protein.
Transcriptional regulation							
19	gi|323307055	Ino4p	11.83/6.59	92	14.42 ± 8.18	245	Transcription factor required for derepression of inositol-choline-regulated genes involved in phospholipid synthesis.
78	gi|323334029	Lsm6p	14.66/9.40	100	10.37 ± 1.77	305	One of the Sm-like proteins; part of heteroheptameric complexes (Lsm2p-7p and either Lsm1p or 8p), involved in RNA degradation.
110	gi|7546208	Prp18p	19.65/9.27	80	13.79 ± 5.11	246	Splicing factor involved in the positioning of the 3′ splice site during the second catalytic step of splicing, part of snRNP U5, interacts with Slu7p.
306	gi|207344589	Ndt80-like protein	53.66/7.60	45	11.45 ± 1.23	141	DNA binding, sequence-specific DNA binding transcription factor activity.
473	gi|256273812	Dbp2p	61.29/8.90	42	13.29 ± 4.54	163	ATP-dependent RNA helicase of the DEAD-box protein family; involved in mRNA decay and rRNA processing.
571	gi|151945667	SCY_5111	64.90/7.90	50	50.27 ± 16.62	195	Hypothetical protein, regulation of transcription.
655	gi|323335109	Aep3p	65.27/9.71	33	23.13 ± 2.72	134	Protein that may facilitate use of unformylated tRNA-Met in mitochondrial translation initiation; stabilizes the bicistronic AAP1-ATP6 mRNA.
789	gi|2131081	Scp160p	93.04/5.46	30	32.14 ± 13.20	162	Essential RNA-binding G protein effector of mating response pathway, mainly associated with nuclear envelope and ER, interacts in mRNA-dependent manner with translating ribosomes via multiple KH domains, similar to vertebrate vigilins.
Amino acid metabolism							
667	gi|6324253	Mks1p	65.72/9.36	51	13.38 ± 2.67	284	Pleiotropic negative transcriptional regulator involved in Ras-CAMP and lysine biosynthetic pathways and nitrogen regulation; involved in retrograde (RTG) mitochondria-to-nucleus signaling.
751	gi|323308391	Gsh1p	77.80/5.87	40	13.33 ± 3.50	187	Gamma glutamylcysteine synthetase.
1005	gi|6320332	Aro1p	175.94/5.90	20	10.41 ± 4.22	74	Pentafunctional arom protein, catalyzes steps 2 through 6 in the biosynthesis of chorismate, which is a precursor to aromatic amino acids
Other proteins							
14	gi|323333563	Rts3p	11.62/10.83	99	32.83 ± 5.19	298	Putative component of the protein phosphatase type 2A complex.
52	gi|259150352	EC1118_1P2_5424p	12.12/9.41	68	18.76 ± 3.51	151	Similar to YPR195C Dubious open reading frame.
83	gi|323305205	Pac2p	55.98/9.17	25	10.76 ± 3.74	70	Microtubule effector required for tubulin heterodimer formation, binds alpha-tubulin, required for normal microtubule function.
85	gi|74583851	Irc11p	17.98/8.76	67	10.48 ± 0.46	256	Putative increased recombination centers protein 11.
126	gi|151945809	SCY_4911	21.99/5.62	34	18.11 ± 7.27	111	Hypothetical protein, participates in carbohydrate metabolic process.
175	gi|151945974	Ppa2p	35.79/6.47	33	10.44 ± 2.10	90	Mitochondrial inorganic pyrophosphatase.
200	gi|6323857	Rad14p	43.47/6.91	27	14.52 ± 3.73	109	Protein that recognizes and binds damaged DNA during nucleotide excision repair; subunit of Nucleotide Excision Repair Factor 1 (NEF1).
279	gi|349581962	Tif3p	48.52/5.12	46	11.23 ± 5.06	201	Nucleic acid binding, nucleotide binding.
329	gi|207343593	MYO3p-like protein	16.49/4.12	67	12.42 ± 4.78	252	Contains one SH3 domain.
346	gi|256273760	Hem14p	60.05/9.33	49	13.77 ± 2.60	202	Protoporphyrinogen oxidase.
940	gi|151944281	Pik1p	120.39/6.02	28	11.12 ± 5.13	148	Phosphatidylinositol 4-kinase; may control nonselective autophagy and mitophagy through trafficking of Atg9p
991	gi|171789	Ypk2p	129.51/9.53	22	11.48 ± 2.06	75	Protein kinase 2, participates in a signaling pathway required for optimal cell wall integrity; homolog of mammalian kinase SGK.

^a^ Spot number refers to Figure 2. ^b^ Coverage of protein sequence by the peptides used for spot identification. ^c^ The fold change of a protein is the spot abundance in the WT divided by that in Pep4p-deficient mutant, only indicated when the difference was statistically significant by more than 10-fold.

## Supplementary Materials

The following are available online at https://www.mdpi.com/2076-2607/7/8/214/s1.
