# Supplementary material for: Identification of Down-Regulated Proteome in Saccharomyces cerevisiae with the Deletion of Yeast Cathepsin D in Response to Nitrogen Stress
Source: Microorganisms. 2019 Jul 24;7(8):214. doi: 10.3390/microorganisms7080214 (PMC6723583; doi:10.3390/microorganisms7080214)
Supplement: Supplementary file 1 [file microorganisms-07-00214-s001.pdf]

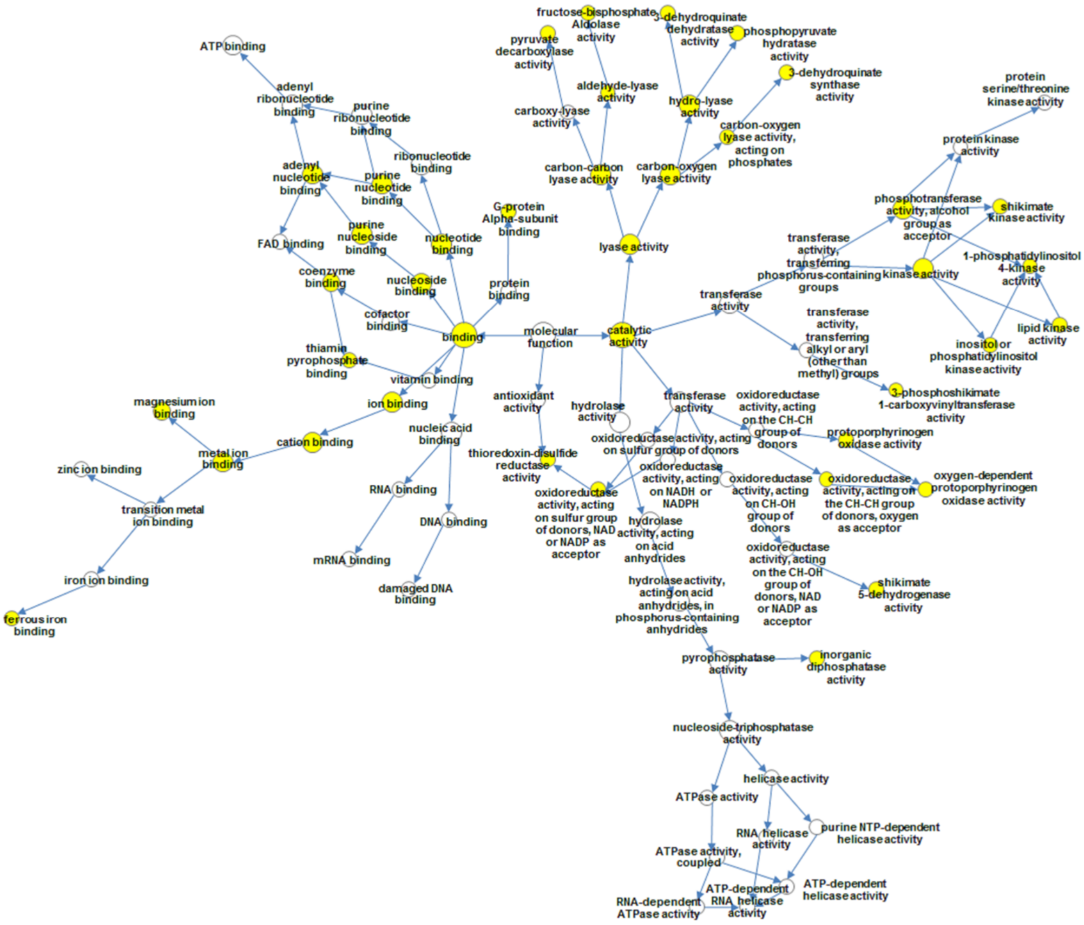

Figure S1. The mass spectrum of spot 187, 188, 208 and 548 in 2-DE gel map of the wild-type yeast strain BY4741.

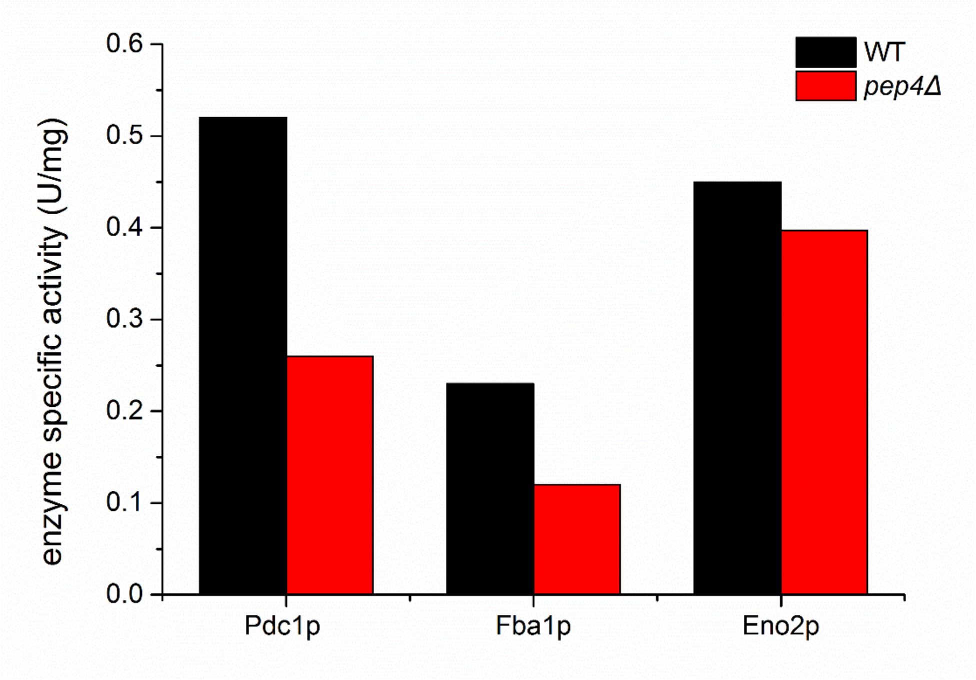

Figure S2. Enzyme specific activity of three identified down-regulated glycolytic metabolism enzymes in pep4Δ strain comparing with the BY4741 (WT). The data represent means ± S.D. (n = 3). * $p < 0.05$, ** $p < 0.01$ compared to BY4741.
